# Warm and Cold Autoimmune Hemolytic Anemia in the Setting of COVID-19 Disease

**DOI:** 10.7759/cureus.18127

**Published:** 2021-09-20

**Authors:** Danielle Brazel, Tarek Eid, Cameron Harding

**Affiliations:** 1 Medicine, University of California Irvine Medical Center, Orange, USA; 2 Internal Medicine, University of California, Irvine, Orange, USA

**Keywords:** cold agglutinins, warm autoimmune hemolytic anemia, mixed autoimmune hemolytic anemia, autoimmune hemolytic anemia (aiha), covid 19

## Abstract

Known associations with autoimmune hemolytic anemia (AIHA) include lymphoproliferative neoplasms, autoimmune conditions, and viral infections. There are a few case reports that implicate a potential relationship between COVID-19 and either warm or cold AIHA. We present the case of combined warm and cold AIHA in the setting of COVID-19. A 51-year-old male with no known past medical history presented with weakness and jaundice. Initial workup revealed white blood cells 41.4, hemoglobin 3.1, platelets 343, total bilirubin 5.3, direct bilirubin 1.6, and COVID-19 positive. Direct antiglobulin test (DAT) found IgG and C3 antibodies and pathology revealed cold agglutinins, consistent with both warm and cold AIHA. He received a total of five blood transfusions and was started on prednisone 1 mg/kg daily with a gradual taper over months. Hemolysis labs normalized within two weeks after discharge although antibodies remained positive 70 days after admission. Our patient presented with IgG and C3 antibodies as well as cold agglutinins, consistent with both warm and cold AIHA. To our knowledge, this is the first case of both warm and cold AIHA presenting simultaneously in COVID-19 infection. Unlike most cases in the existing literature, this patient had no history of underlying hematologic malignancy and both warm and cold AIHA.

## Introduction

Coronavirus-19 (COVID-19) was declared a global pandemic in March 2020 and by April 2021 resulted in over 30 million positive tests and 555,000 deaths within the United States [1]. COVID-19 clinical course ranges from asymptomatic to fulminant respiratory failure.

Warm autoimmune hemolytic anemia (AIHA) is caused by IgG binding to red blood cells mediating phagocytosis or destruction by the reticuloendothelial system [2,3]. It is associated with lymphoproliferative diseases (chronic lymphocytic leukemia, non-Hodgkin lymphoma), autoimmune diseases (systemic lupus erythematosus and rheumatoid arthritis), infections (HIV), and medications (penicillin and cephalosporin) [3]. Cold AIHA is caused by IgM binding to the RBC membrane and activating complement, which leads to intravascular hemolysis or phagocytosis in the extravascular space [4]. It is associated with infections (Mycoplasma pneumoniae, hepatitis C, Epstein-Barr virus, and cytomegalovirus), autoimmune disease, and B-cell lymphoproliferative diseases [5,6].

An increasing body of evidence confirms that severe cases of COVID-19 infection cause a hyperinflammatory syndrome and cytokine storm [7]. Several case reports and case series have implicated an association between COVID-19 infection and either warm or cold AIHA [7-11]. We present simultaneous onset of COVID-19 and both warm and cold AIHA in a patient with no medical comorbidities.

## Case presentation

A 51-year-old undoctored male with no known past medical history presented with weakness and jaundice. Approximately one week prior to presentation, the patient developed intermittent fevers and confusion. Two days prior to presentation, he developed fatigue, jaundice, and dark urine. He had one episode of non-bloody emesis but denied hematemesis, melena, hematochezia, or pain. He denied personal or family history of sickle cell disease, thalassemia, or malignancies.

**Figure 1:**
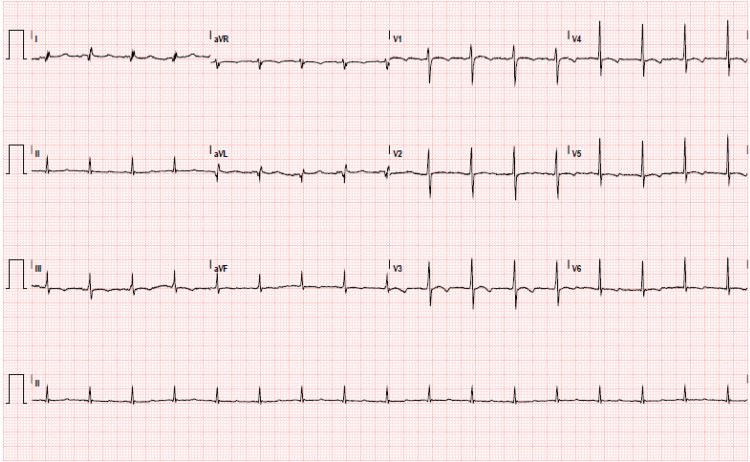
Electrocardiogram (EKG) showing supraventricular tachycardia with nonspecific ST-segment and T-wave changes

**Figure 2:**
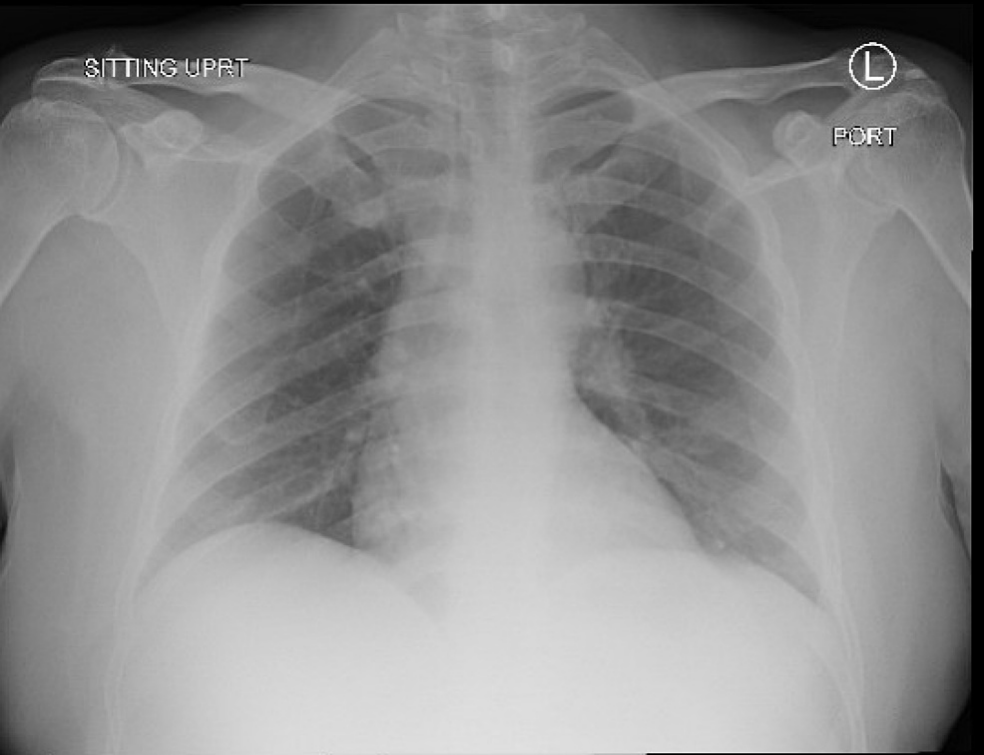
Initial chest radiograph showing no focal evidence of airspace disease

**Figure 3:**
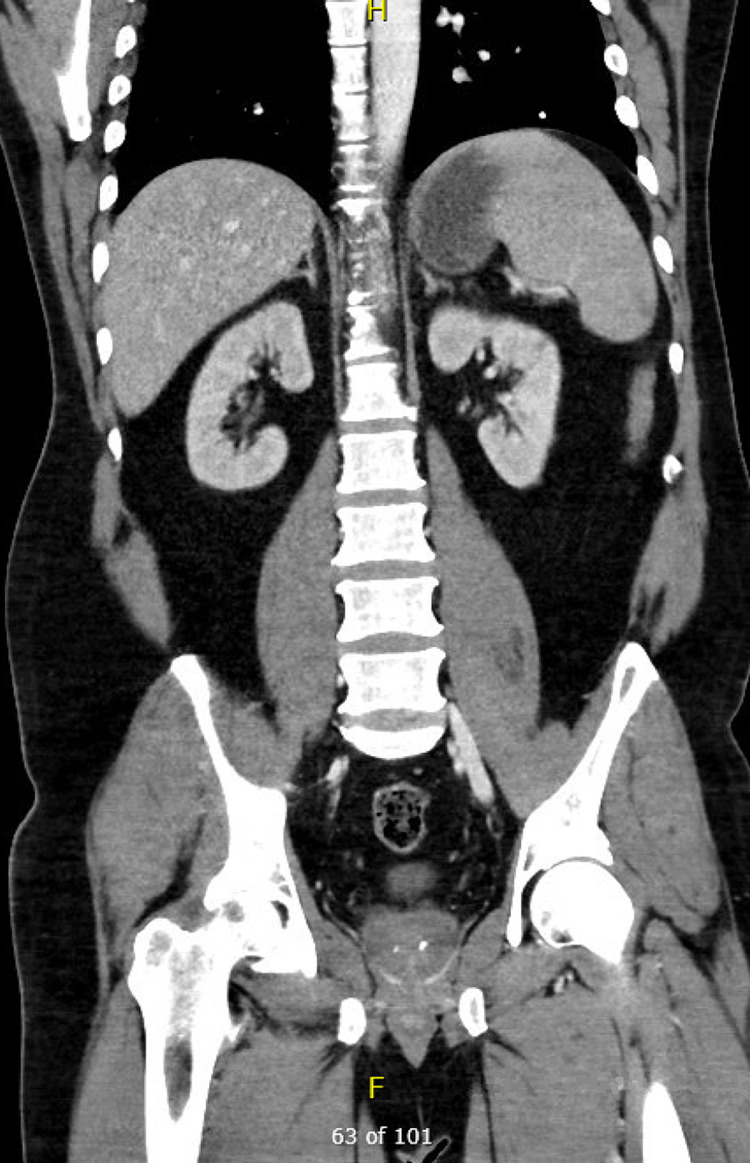
CT abdomen and pelvis showing mild splenomegaly

On arrival, he was afebrile with heart rate of 114, blood pressure 110/61, respiratory rate 18, oxygen saturation 97% on 5 liters via nasal cannula. Initial workup revealed white blood cells 41.4 thousand cells/MCL, hemoglobin 3.1 g/dL, MCV 124.1 FL, platelets 343 thousand cells/mL, creatinine 1.4 mg/dL, total bilirubin 5.3 mg/dL, direct bilirubin 1.6 mg/dL, and COVID-19 positive by reverse transcription-polymerase chain reaction testing. Electrocardiogram (EKG) revealed supraventricular tachycardia with nonspecific ST segment and T wave changes (Figure 1). A chest radiograph showed no focal evidence of airspace disease (Figure 2) and CT abdomen and pelvis revealed hepatic steatosis and mild splenomegaly (Figure 3). Four units of packed red blood cells were transfused with response in hemoglobin to 6.8.

**Figure 4:**
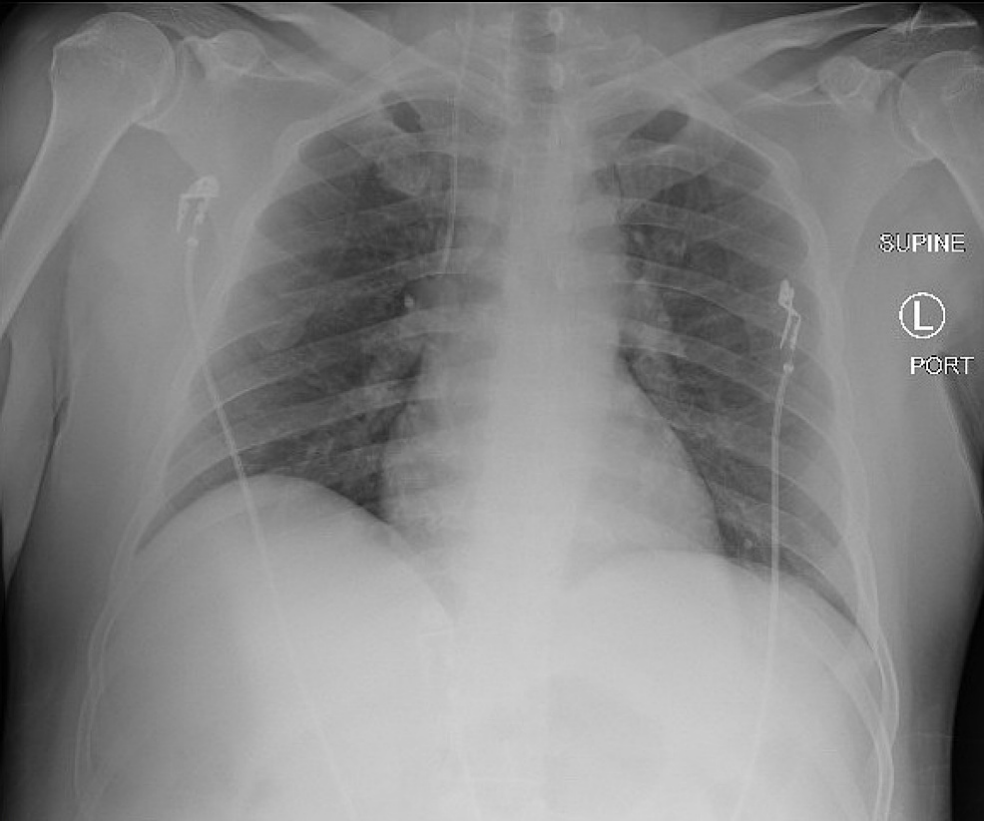
Repeat chest x-ray showed bibasilar opacities worse throughout the right lobes

Peripheral blood smear found primarily nucleated red blood cells, no abnormal white blood cells, and no increase in lymphocyte quantity. Further workup showed iron studies, B12, and folate within normal ranges. COVID-19 severity labs included lactate dehydrogenase (LDH) 1460 U/L, fibrinogen 488 mg/dL, d-dimer 1780 ng/mL, erythrocyte sedimentation rate (ESR) 130 mm/hr, C-reactive protein (CRP) 4.44 mg/dL, and procalcitonin 1.43 ng/dL (Table 1). Initial hematologic analysis from hospital day 1 showed blood type A+, Rh+. Direct antiglobulin test (DAT) was positive for IgG and C3 complement antibodies, consistent with warm AIHA. Hematopathologist review also revealed agglutination on immediate spin reactivity consistent with cold AIHA. On hospital day 2, hemoglobin returned at 6.6 and he was given the fifth unit of blood. His creatinine normalized to 0.8 with fluids. Infectious workup including hepatitis B, hepatitis C, and HIV returned negative. Repeat chest x-ray showed bibasilar opacities worse throughout the right lobes (Figure 4). Flow cytometry found 0.4% circulating myeloblasts without evidence for malignancy. He was started on the empiric treatment of AIHA with prednisone 1 mg/kg daily (100 mg daily).

**Table 1:** Relevant Laboratory Values WBC: white blood cell; Hgb: hemoglobin; Plt: platelet; T Bili: total bilirubin; LDH: lactate dehydrogenase; Coombs IgG: Coombs test immunoglobulin G

	Day 1	Day 2	Day 3	Day 4	Day 5	Day 6	Day 7	Day 8	Day 24 follow-up	Day 70
WBC	41.4	26.9	19.2	16.4	12.2	8.1	7.1	6.8	11.0	8.7
Hgb	3.1	6.6	8.0	7.9	8.2	7.9	8.2	8.7	13.8	14.0
Plt	343	248	151	112	82	65	68	74	367	
T Bili	5.3	5.4	3.5	2.9	2.2	1.9	1.6	1.7	0.8	0.4
Haptoglobin		<30						<30	72	81
LDH	1394	1460	1363		1097			681	237	232
Reticulocyte count	14.2%							13.9%	3.4%	
Coombs IgG	2+ positive								2+ positive	1+ positive
Anti-complement	2+ positive								Weak positive	

His platelet count decreased throughout his admission with a low of 65 on hospital day 6. Heparin antibody and serotonin release assay both returned negative for heparin-induced thrombocytopenia. On hospital day 3, CT chest with pulmonary embolus protocol showed multiple segmental and subsegmental pulmonary emboli in all lobes, few occlusive but mostly nonocclusive with moderate clot burden. He was started on apixaban.

He was discharged on hospital day 8 on prednisone 100 mg daily. Repeat flow cytometry performed two weeks after discharge was normal. His hemolysis labs including hemoglobin, bilirubin, haptoglobin, and LDH normalized by his two-week follow-up appointment. Prednisone was decreased to 80 mg daily at one month post-discharge and 60 mg daily at two-month follow-up. At his most recent follow-up 70 days since admission, DAT for IgG and anti-complement remained positive and prednisone was decreased to 50 mg daily.

## Discussion

Our patient presented with fatigue, jaundice, severe anemia, elevated LDH, and decreased haptoglobin indicative of hemolytic anemia. This case demonstrates both warm and cold AIHA confirmed by DAT and agglutination on immediate spin testing on hospital day 1. In immediate spin testing, the patient’s plasma and donor red cells are spun without the addition of antiglobulin. IgG is not large enough to agglutinate on a rapid spin. This indicates that a precipitate without additives below physiologic temp is diagnostic of IgM disease such as cold AIHA. Our institution uses immediate spin reactivity as a cold agglutinin screen. Thermal amplitude testing is not performed at our institution. Hemolysis labs including hemoglobin, bilirubin, haptoglobin, and LDH normalized two weeks after discharge but antibodies remain positive even through 70-day follow-up. He was started on prednisone 1 mg/kg daily (100 mg daily) and gradually tapered down to 50 mg by a two-month follow-up. Hematology considered the addition of rituximab and bendamustine for cold AIHA but deferred when our patient dramatically improved on steroids.

Unlike most existing cases in the literature, our patient had no underlying hematologic malignancy, autoimmune disease, or predisposing factors for high-risk COVID-19 infection. In a recent review of seven patients with AIHA associated with COVID-19, five had underlying malignancy [7]. This case series reported four cases with warm antibodies and three with cold agglutinins. Five patients were treated with corticosteroids and two required red blood cell transfusions. Another case report treated a patient with simultaneous COVID-19 and AIHA with intravenous immunoglobulin (IVIG) and, consistent with prior literature showing poor response to IVIG in AIHA, later started the patient on prednisone and blood transfusions [10].

The current case found declining platelets with a nadir of 65 on hospital day 6. The etiology of his thrombocytopenia was unclear though suspected due to his underlying COVID-19 infection or associated immune thrombocytopenic purpura (ITP). The patient was evaluated for heparin-induced thrombocytopenia (HIT) but both serotonin release assay and heparin antibody were negative. Thrombotic thrombocytopenic purpura (TTP) was also considered but determined to be less likely. ITP is associated with viral infections including hepatitis B, hepatitis C, cytomegalovirus, varicella-zoster, human immunodeficiency virus, zika, and COVID-19 [12]. A recent systematic review of 45 cases of ITP in COVID-19 found the majority (71%) in elderly patients and 75% in moderate-to-severe illness from COVID-19 [13]. Up to 31% did not report bleeding complications at the time of diagnosis, which would be consistent with our patient's case. The authors found a good initial response to a short course of glucocorticoids and intravenous immunoglobulin in all but one case. Given the association between ITP and COVID-19 [14] illness in addition to our patient’s marked improvement after initiation of steroids, ITP is the most likely cause of his thrombocytopenia.

## Conclusions

To date, only a few case reports and one case series describe patients presenting with COVID-19 and AIHA simultaneously. Although the association between COVID-19 and coagulopathies has been well described, future studies are needed to determine whether a causal relationship exists between COVID-19 and AIHA. Clinicians should use caution in patients with declining hemoglobin levels knowing that hemolysis may be masked in COVID-19 infection by frequently elevated LDH and elevated acute phase haptoglobin. Given that both COVID-19 and cold agglutinin hemolytic anemia increase the risk of thrombotic events and DIC, more research on optimal treatment is necessary to prevent poor outcomes.
